# Postbleeding antithrombotic management in cardiovascular patients: insights from the Attica Bleeding Snapshot study

**DOI:** 10.1016/j.rpth.2026.106864

**Published:** 2026-07-15

**Authors:** Stergios Soulaidopoulos, Ioannis Leontsinis, Christina Chrysochoou, Eirini Dri, Fotios Tatakis, Ioannis Kyriazis, Elias Skopelitis, Vasilios Sevastianos, Eleni Korompoki, Aikaterini Touliatou, Konstantina Paraskeva, Natalia G. Vallianou, Sotirios Tsiodras, Amanda Psyrri, Spilios Manolakopoulos, George Papatheodoridis, Dimitrios Vassilopoulos, Nikolaos Tentolouris, Konstantinos Tsioufis, Centers and Investigators, M. Dimopoulos, M. Gavriatopoulou, A. Mitrakou, D. Sinapidis, E. Kostis, E. Kerezoudi, S. Michopoulos, N. Kontomitros, S. Pililis, A. Fragkou, S. Lagou, L. Lemonidis, K. Vrysis, P. Kalisperati, T. Kontopoulou, I. Iliopoulos, G. Pournaras, V. Sevastianos, P. Stoikos, E. Geladari, G. Markomichelakis, P. Efthimiou, D. Katsavarou, V. Papastamopoulos, A. Siampanos, N. Petridis, M. Chroni, N. Viazis, G. Adamis, A. Touliatou, C. Valilas, A. Kougioumtzoglou, G. Grigoriou, E. Tzintziloni, S. Pagoni, I. Karoumpalis, G. Boulmetis, A. Argyriadi, G. Kourmpelis, G. Aravantinos, I. Mani, I. Kyriazis, A. Apostolos, C. Lampropoulos, E. Koukou, V. Argiana, S. Zacharioudaki, A. Tzavaras, E. Kaldara, K. Katsiofoti, G. Tziatzios, P. Sfikakis, E. Apostolopoulou, E. Gkogka, M. Samarkos, M. Voulgarelis, E. Apostolidi, D. Germanou, E. Michelakis, A. Vakka, S. Gaitanou, S. Fragkiski, S. Menegakis, E. Michaliou, A. Liapi, E. Agelonidou, K. Souvatzi, A. Krilis, E. Diamantaki, A.M. Xirikou, V. Tzavara, A. Lioni, V. Lampropoulou, D. Papanikolaou, M. Chini, D. Kostourou, E. Manou, E. Geroniti, G. Trimponias, P. Zormpas, M. Spinou, E. Tzivaki, M. Lada, V. Kolonia, M. Mprinia, E. Lalla, K. Syrigos, A. Kollias, A. Argyraki, T. Katsikas, C. Zirou, G. Mpamias, C. Gkizis, I. Koutsounas, S. Antonopoulos, G. Athanasakis, M. Vroucho, S. Sympardi, E. Kaltsounis, T. Tsampras, K. Iliopoulou, N. Papadopoulos, G. Iliopoulos, A. Katsiki, M. Mpoulougari

**Affiliations:** 1 First Department of Cardiology, Medical School, National and Kapodistrian University of Athens, Hippokration Hospital, Athens, Greece; 2 Internal Medicine Department, KAT General Hospital, Athens, Greece; 3 Third Department of Internal Medicine, General Hospital of Nikaia-Piraeus “Agios Panteleimon”, Athens, Greece; 4 Third Department of Internal Medicine & Liver Unit, Evangelismos General Hospital, Athens, Greece; 5 Department of Clinical Therapeutics, Medical School, National and Kapodistrian University of Athens, Alexandra Hospital, Athens, Greece; 6 Second Department of Internal Medicine, G. Gennimatas General Hospital, Athens, Greece; 7 Department of Gastroenterology, “Konstantopouleio–Patision” General Hospital, Nea Ionia, Athens, Greece; 8 First Department of Internal Medicine, Sismanogleio General Hospital, Athens, Greece; 9 Fourth Department of Internal Medicine, Medical School, National and Kapodistrian University of Athens, Attikon General Hospital, Athens, Greece; 10 Medical Oncology Unit, Second Propaedeutic Department of Medicine, Medical School, National and Kapodistrian University of Athens, Attikon General Hospital, Athens, Greece; 11 GI-Liver Unit, Second Department of Internal Medicine, Medical School, National and Kapodistrian University of Athens, Hippokration Hospital, Athens, Greece; 12 First Department of Gastroenterology, Medical School, National and Kapodistrian University of Athens, “Laiko” General Hospital, Athens, Greece; 13 Second Department of Internal Medicine, Medical School, National and Kapodistrian University of Athens, Hippokration Hospital, Athens, Greece; 14 First Department of Propaedeutic and Internal Medicine, Medical School, National and Kapodistrian University of Athens, “Laiko” General Hospital, Athens, Greece

**Keywords:** antithrombotic therapy, atrial fibrillation, frailty, major bleeding, postbleeding management

## Abstract

**Background:**

Bleeding complications in cardiovascular patients receiving antithrombotic therapy pose a major clinical challenge, particularly given the limited evidence guiding antithrombotic management after a bleeding event.

**Objectives:**

This snapshot study aimed to evaluate the burden of major bleeding events requiring hospitalization and to describe real-world practices in postbleeding antithrombotic management.

**Methods:**

The Attica Bleeding Snapshot was a multicenter, cross-sectional observational study conducted from February 17 to 21, 2025, in the Internal Medicine and Gastroenterology Departments of 17 hospitals in the Attica region, Greece. Adult patients hospitalized for major bleeding while receiving antithrombotic therapy for cardiovascular indications were included. Data collected comprised demographics, comorbidities, antithrombotic regimens, bleeding site, management strategies, and in-hospital outcomes.

**Results:**

Of 1540 hospitalized patients, 134 (8.7%) experienced major bleeding while on antithrombotic therapy. The mean ± SD age was 79.5 ± 11.1 years, and 38.1% were female. Atrial fibrillation was the most frequent indication for antithrombotic therapy (46.2%), followed by coronary artery disease (20.9%). Gastrointestinal bleeding accounted for 63.5% of cases. In-hospital mortality was 14.9%, with frailty independently associated with mortality (odds ratio, 1.64; 95% CI, 1.24-2.16; *P* < .001). Approximately one-third of patients were discharged without antithrombotic therapy, irrespective of ischemic risk.

**Conclusion:**

Major bleeding during antithrombotic treatment is associated with substantial morbidity and mortality in cardiovascular patients. Postbleeding antithrombotic management remains highly heterogeneous, underscoring a critical unmet need. Alternative strategies for thromboembolic prevention, including left atrial appendage occlusion, merit consideration in selected high-risk atrial fibrillation patients, particularly those with recurrent bleeding.

## Essentials

• Major bleeding during antithrombotic treatment is common and can be life-threatening.
• Patients hospitalized for major bleeding in internal medicine and gastroenterology departments across 17 hospitals were enrolled in this multicenter snapshot study.
• During a 1-week study period, major bleeding accounted for 8.7% of all hospitalizations.
• Nearly 15% of patients died, while one-third left the hospital without antithrombotic treatment.

## 1 Introduction

Bleeding events represent a major clinical challenge in the management of patients with cardiovascular disease receiving antithrombotic therapy for primary or secondary prevention. Although antithrombotic therapy plays a fundamental role in thrombotic or thromboembolic risk reduction strategies, it carries an inherent risk of hemorrhagic complications. This risk is further amplified in patients receiving combinations of antithrombotic agents, as well as in those with advanced age or coexisting comorbidities [1].

Epidemiologic data indicate that up to ∼15% of adults in Europe receive antiplatelet and/or oral anticoagulants (OACs) [2]. On the contrary, reported rates of major bleeding among cardiovascular patients range from 0.5% to 1% in those receiving single antiplatelet therapy to as high as 8% to 10% in those treated with triple antithrombotic regimens [3,4]. In the setting of atrial fibrillation (AF) trials, randomized evaluation of long-term anticoagulation therapy reported annual major bleeding rates between 2.7% and 3.4%, with lower rates recorded with dabigatran 110 mg and higher rates with warfarin [5]. Trials of AF patients undergoing percutaneous coronary intervention (PCI) demonstrated that non-vitamin K OACs combined with a single P2Y_12_ inhibitor reduced bleeding compared with traditional triple therapy, yet rates remained substantial, ranging from 10% to 20%, underscoring that dual antithrombotic regimens are also linked to a considerable hemorrhagic risk [6].

When bleeding occurs, decisions regarding continuation, optimal time of restarting, dose adjustment, or temporary cessation of treatment are particularly complex in patients with AF and/or those with recent PCI. Advances in interventional cardiology, including thin-strut drug-eluting stents, drug-coated balloons, and individualized, risk-stratified dual antiplatelet therapy (DAPT) durations, have enhanced the ability to take a personalized approach to the duration and combination of antithrombotic regimens [7,8]. Moreover, interventional transcatheter techniques for left atrial appendage (LAA) occlusion offer an alternative strategy for stroke prevention in AF patients at high bleeding risk [9].

Despite the well-recognized risk of bleeding associated with antithrombotic treatment in cardiovascular patients, real-world data on the burden and management of such events remain limited. Moreover, clinical practice regarding the resumption of antithrombotic therapy varies considerably, reflecting the absence of clear, evidence-based guidelines. Our study sought to address these gaps through a multicenter snapshot survey conducted during a prespecified period in internal medicine and gastroenterology departments across the Attica region in Greece, aiming to capture real-world patterns of care and highlight areas of uncertainty in postbleeding antithrombotic management.

## 2 Methods

The snapshot took place across internal medicine and gastroenterology departments in the Attica region of Greece between February 17 and 21, 2025, under the auspices of the First Department of Cardiology of the Medical School, National and Kapodistrian University of Athens.

All internal medicine and gastroenterology departments in the Attica region, which encompasses the entire Athens metropolitan area, were invited to participate and enroll patients in the snapshot study. Eligible patients were those hospitalized for a bleeding event within the study timeframe while receiving antithrombotic therapy for a cardiovascular indication. Patients already hospitalized for bleeding at the onset of the snapshot and those admitted to participating centers during the study period were included. Major bleeding was defined as any event that required hospital admission; minor bleeding events managed in the emergency department or in the outpatient setting were excluded. Standard bleeding classifications (eg, International Society on Thrombosis and Haemostasis [ISTH] major bleeding) were not applied, as the study focused exclusively on clinically significant bleeding episodes necessitating inpatient management. Deanonymized data were collected and entered into a prespecified electronic case report form. This form was created through the online REDCap platform (Vanderbilt University), which enabled simultaneous and secure data entry.

Deanonymized data included patients’ demographics, physical characteristics, and lifestyle parameters. Information regarding the patients’ medical history, focusing on cardiovascular history and indication for antithrombotic medication, was recorded. Moreover, each participating center collected data on bleeding episodes, such as the site of bleeding, the precise antithrombotic regimen the patients were on, the cardiac rhythm on electrocardiogram at the time of admission, the need for blood transfusion, the way the hemorrhage was managed, the total days of hospitalization, and the patient’s final clinical outcome. For each enrolled patient, frailty was assessed using the Rockwood Clinical Frailty Scale, a validated 9-point global clinical scale, with higher scores indicating increasing frailty and greater vulnerability to adverse outcomes.

The study protocol was in accordance with the Declaration of Helsinki and was approved by the local Ethical Committees of each participating institution.

### 2.1 Statistical analysis

Data were analyzed using SPSS version 29.0.2.0 (IBM Corporation). Categorical variables are presented as percentages. Continuous variables that are normally distributed are expressed as means ± SDs, while those that are nonnormally distributed are expressed as medians and ranges. The Kolmogorov–Smirnov test was used to assess the normality of the distribution of continuous variables. Comparisons between continuous variables were performed using a two-sample *t*-test when normally distributed, or the Mann–Whitney U-test. Differences between categorical variables were assessed with the chi-squared test. Univariable logistic regression was used to assess factors associated with discontinuation of antithrombotic therapy at discharge and mortality. Variables with a *P* value <.10 in univariable analysis were subsequently entered into a multivariable logistic regression model to identify independent predictors of mortality. A two-tailed *P* value <.05 was considered statistically significant for all tests.

## 3 Results

### 3.1 Demographics and medical history

Seventeen hospitals in the Attica region of Greece, including 24 internal medicine departments and 5 gastroenterology departments/clinics, participated by enrolling patients in the Attica Bleeding Snapshot study. A total of 1540 patients were hospitalized in the recruiting departments during the study period. Notably, 134 of these patients were admitted due to major bleeding and were enrolled in the study, representing 8.7% of all hospitalized patients. Their mean ± SD age was 79.5 ± 11.1 years, and women accounted for 38.1% of the total sample.

Almost half of the included patients (*n* = 61, 45.6%) were on antihypertensive treatment at admission, and 30.6% (*n* = 41) had diabetes mellitus. Additionally, 1 of 5 patients had been diagnosed with heart failure, 9% with chronic kidney disease, and 17.9% had a history of ischemic stroke. With respect to a history of bleeding, 7.5% of patients had a history of major gastrointestinal (GI) bleeding, 0.7% a history of hemorrhagic stroke, and 4.5% a history of bleeding at another site.

**Figure 1:**
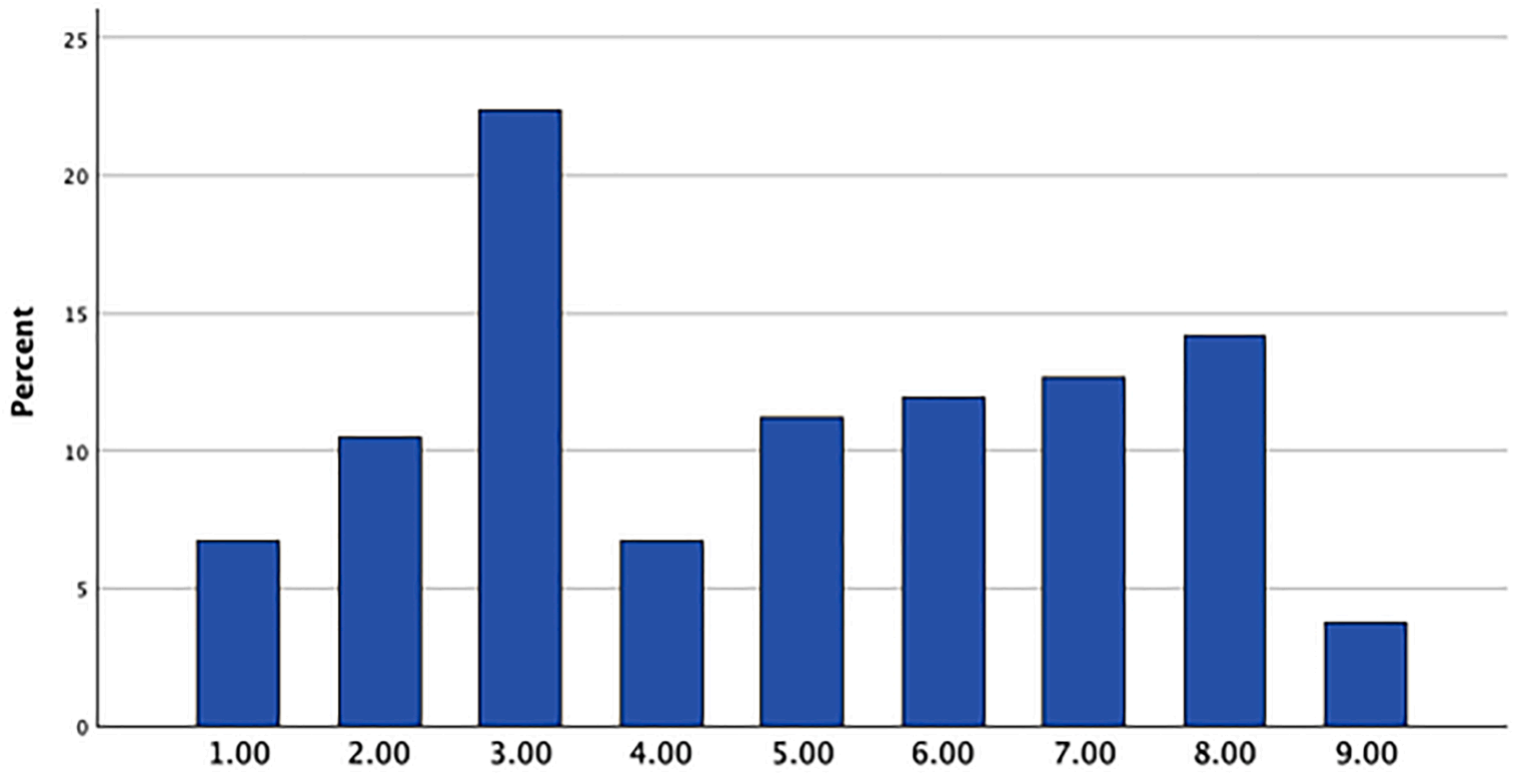
Classification of patients based on the Rockwood Frailty Scale.

The mean ± SD Rockwood Frailty Scale score in our sample was 4.8 ± 2.3, with the majority of patients classified as “managing well” (score 3; 22.5%). Classification of the sample according to the Rockwood Frailty Scale is illustrated in Figure 1.

**Figure 2:**
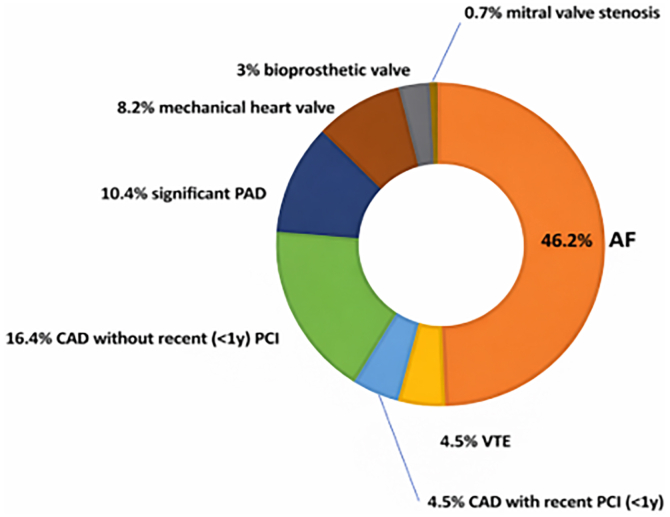
Indication for antithrombotic treatment among the study population. AF, atrial fibrillation; CAD, coronary artery disease; PAD, peripheral arterial disease; PCI, percutaneous coronary intervention; VTE, venous thromboembolism.

The most frequent indication for antithrombotic treatment was AF, either chronic or paroxysmal. Specifically, 46.2% of patients (*n* = 62) were receiving anticoagulation therapy for AF: 36.6% with chronic AF and 9.6% with paroxysmal AF. Coronary artery disease was indicated in 20.9% of patients (*n* = 28); 16.4% were on antithrombotic treatment without a recent PCI, while 4.5% were receiving treatment following a recent coronary intervention (<1 year). One patient presenting with upper GI bleeding had a history of PCI performed more than a year ago and was still on DAPT. Other indications for antithrombotic treatment included significant peripheral arterial disease (10.4%), the presence of a mechanical heart valve (8.2%), a history of venous thromboembolism (4.5%), and the presence of a bioprosthetic heart valve (3%). Figure 2 schematically illustrates the indications for antithrombotic treatment among patients who experienced a hemorrhagic event and were included in the study.

### 3.2 Data on bleeding events and outcomes

**Figure 3:**
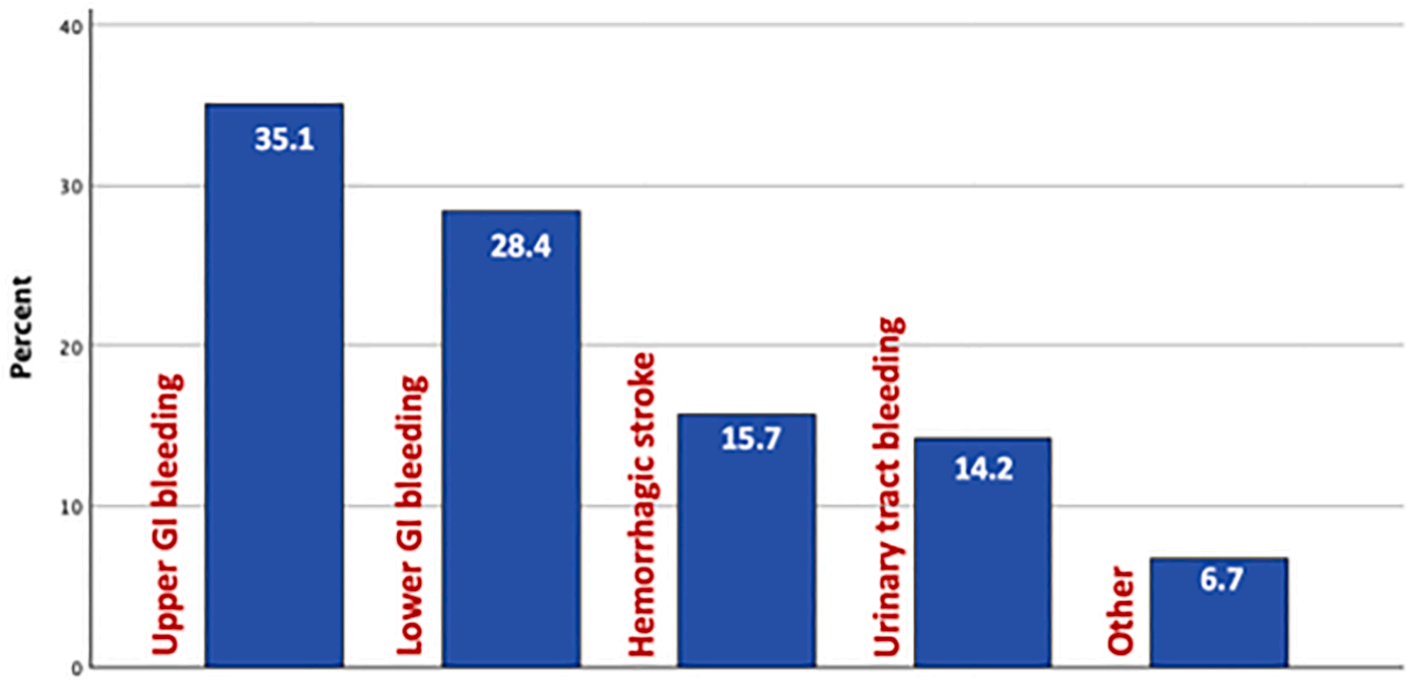
Sites of bleeding (%) among the study population. GI, gastrointestinal.

The mean ± SD time of hospitalization for the 134 patients included in the snapshot study was 10.2 ± 11.6 days, with a median of 7 days. Twenty-nine patients were hemodynamically unstable on hospital arrival. Regarding the site of bleeding, 35.1% of patients (*n* = 47) experienced upper GI bleeding, 28.4% (*n* = 38) had lower GI bleeding, 15.7% (*n* = 21) were diagnosed with hemorrhagic stroke, and 14.2% (*n* = 19) were hospitalized due to urinary tract bleeding. In 6% of cases, the bleeding occurred at other sites (Figure 3).

**Figure 4:**
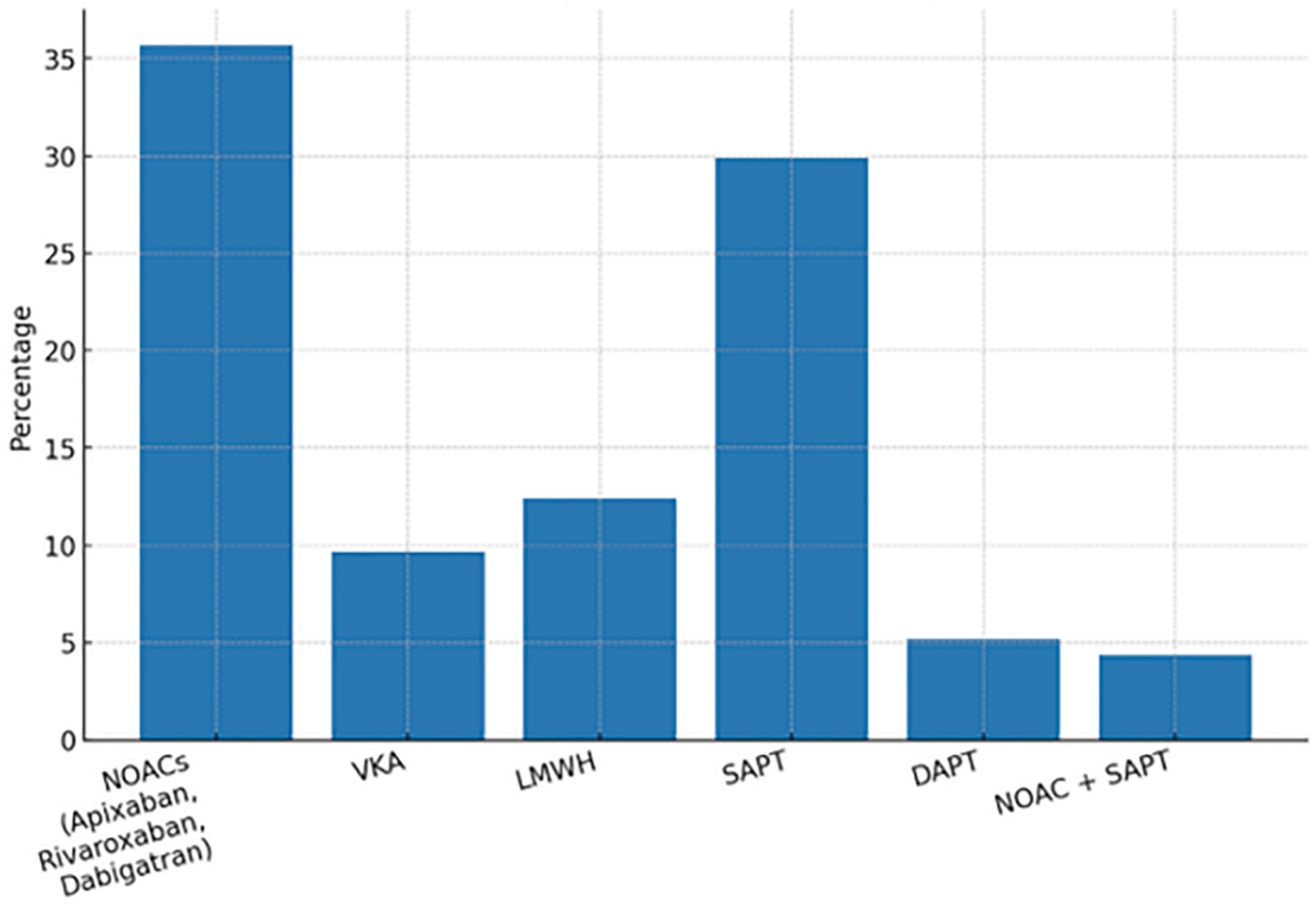
Distribution of antithrombotic regimens (%) among the study population. DAPT, dual antiplatelet therapy; LMWH, low-molecular-weight heparin; NOAC, new oral anticoagulant; SAPT, single antiplatelet therapy; VKA, vitamin K antagonist.

With respect to the antithrombotic regimen, 35.7% of patients (*n* = 48) received new OACs (NOACs; 21.6% [*n* = 29] apixaban, 9.7% [*n* = 13] rivaroxaban, and 4.4% [*n* = 6] dabigatran), 9.7% (*n* = 13) vitamin K antagonists, 12.7% (*n* = 17) low-molecular-weight heparin (LMWH), 29.9% (*n* = 40) single antiplatelet therapy (27 aspirin and 13 clopidogrel monotherapy), and 5.2% (*n* = 7) DAPT (6 aspirin and a clopidogrel combination [1 aspirin and a ticagrelor]). Six patients were treated with combination therapy with a NOAC (5 apixaban and 1 rivaroxaban) and a single antiplatelet agent; however, only 2 of these patients had AF and a history of recent coronary PCI (Figure 4). At the time of the bleeding episode, 3% of patients were receiving concomitant treatment with nonsteroidal anti-inflammatory drugs, and 3.7% were treated with steroids. The majority of patients were on gastroprotective therapy prior to the bleeding event. Specifically, 64.9% of patients (*n* = 87) were on chronic treatment with proton pump inhibitors, 1.5% were receiving H2 receptor antagonists, and 33.6% (*n* = 45) were not on any gastroprotective medication. Nevertheless, 19 patients presenting with upper GI bleeding did not receive gastroprotection, representing 40.4% of all upper GI bleeding cases.

Bleeding was managed conservatively in 47% of patients (*n* = 63). Transfusion of red blood cells was required in 53% of patients (*n* = 71), while fresh frozen plasma was administered in 16.4% (*n* = 22). Adexanet A was administered in 2.2% of patients and vitamin K in 3%. An endoscopic procedure was required in 14.9% of patients to control bleeding; all were performed for GI bleeding, while a surgical procedure was necessary in 1.5% of patients for the same reason.

Of the 134 patients hospitalized for a bleeding event, 20 died, corresponding to an in-hospital mortality rate of 14.9%. In univariate analysis, the Rockwood Frailty Scale score and prophylactic LMWH dosage were associated with increased mortality (odds ratio [OR], 1.63, *P* < .001; OR, 4.04, *P* = .047, respectively), while hemodynamic instability at admission showed a nonsignificant trend toward higher mortality (OR, 2.75; *P* = .06). In the subsequent multivariable logistic regression analysis, only the Rockwood Frailty Scale score remained independently associated with mortality. Specifically, each 1-point increase in the frailty score was associated with a 64% higher risk of death (OR, 1.64; 95% CI, 1.25-2.16; *P* < .001).

The majority of patients (*n* = 98, 73.1%) achieved clinical stabilization and were discharged. Regarding antithrombotic therapy at discharge, 11 patients were prescribed apixaban 2.5 mg twice daily, 7 apixaban 5 mg twice daily, 4 rivaroxaban 15 mg once daily, 1 dabigatran 110 mg twice daily, 1 dabigatran 150 mg twice daily, 7 vitamin K antagonists, 12 aspirin 100 mg, 11 clopidogrel, and 22 LMWH, while 34 (29.8%) received no antithrombotic treatment. Furthermore, 4 patients were discharged on DAPT, compared with 7 at admission.

**Figure 5:**
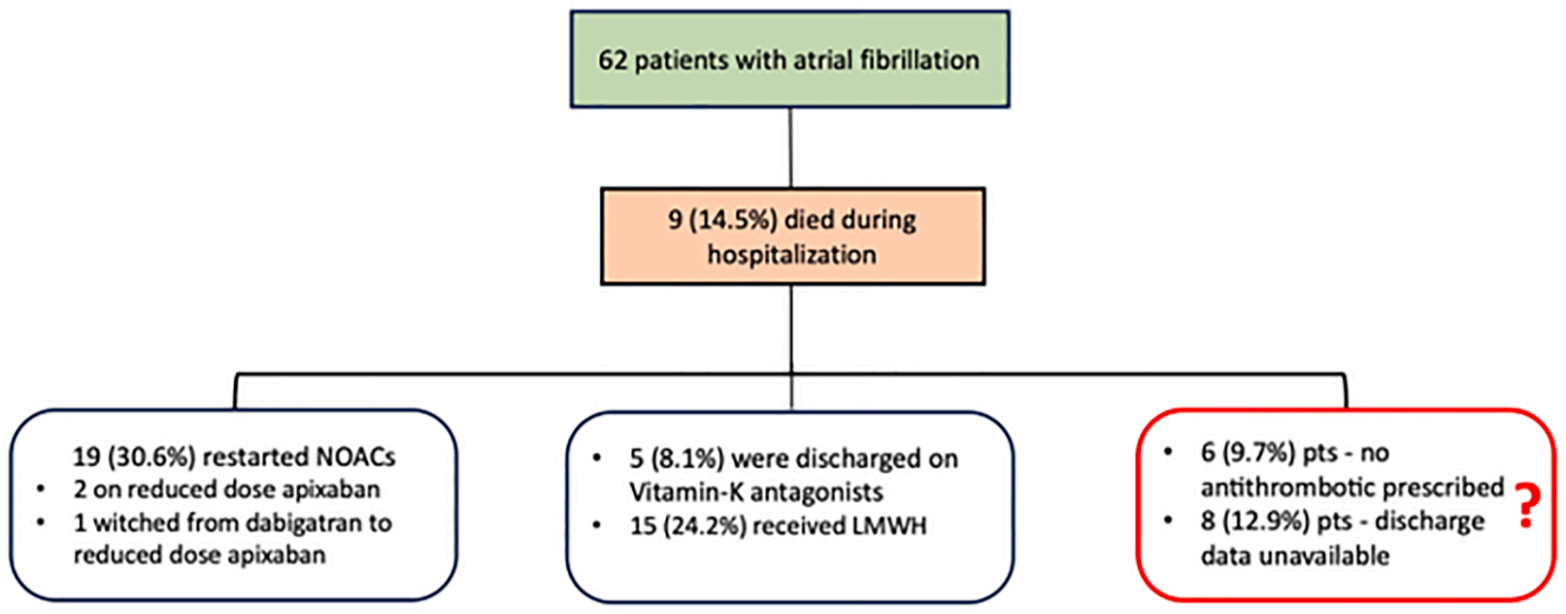
Discharge antithrombotic management for patients with atrial fibrillation. LMWH, low-molecular-weight heparin; NOAC, new oral anticoagulant; pts, points.

Focusing on patients with AF, 9 died during hospitalization (14.5%, 9/62). Among the survivors, 19 resumed NOAC therapy, including 2 treated with a reduced dose of apixaban and 1 who switched from dabigatran to apixaban 2.5 mg. Six patients were discharged without any antithrombotic therapy, while 15 received LMWH and 5 were discharged on vitamin K antagonists. For 8 patients, discharge antithrombotic management was not documented and could not be classified (Figure 5).

Overall, 34 patients (29.8%) were discharged home without antithrombotic therapy. In univariable logistic regression analysis, discontinuation of antithrombotic therapy at discharge was significantly associated with AF as the indication for anticoagulation at admission (*P* = .01) and with the absence of any absolute indication for antithrombotic treatment at admission (*P* < .001). Notably, 6 of the 53 surviving patients with AF and 11 without a clear indication for antithrombotic therapy were discharged without any pharmacologic prevention of ischemic events.

## 4 Discussion

In this multicenter snapshot study conducted across internal medicine and gastroenterology departments of the Attica region, the outcomes were as follows:

i. Major bleeding accounted for 8.7% of all hospitalized admissions during the study period among patients receiving antithrombotic therapy.
ii. Bleeding events were associated with substantial morbidity and mortality, with a mean hospital stay of 10 days and an in-hospital mortality rate of 14.9%.
iii. Nearly half of bleeding-related hospitalizations occurred in patients receiving OACs for AF, identifying this group as a major contributor to bleeding burden.
iv. Approximately one-third of patients were discharged without any antithrombotic therapy, underscoring significant heterogeneity and uncertainty in postbleeding management.
v. GI bleeding was the most frequent presentation, followed by hemorrhagic stroke and urinary tract bleeding; notably, almost half of patients with upper GI bleeding were not receiving gastroprotective therapy.

Unlike previous studies focusing mainly on bleeding incidence in cardiovascular cohorts, this snapshot provides a real-world view of hospitalized patients managed in routine internal medicine practice. Bleeding is a frequent complication of antithrombotic therapy, particularly with DAPT or anticoagulation, and bleeding risk increases with treatment intensity and duration. Importantly, bleeding events are often managed by physicians different from those who initiated antithrombotic therapy, underscoring that this is not only a cardiovascular issue but a broader healthcare challenge.

Overall, GI bleeding accounted for nearly two-thirds of cases, and notably, 40.4% of patients with upper GI bleeding were not receiving gastroprotective therapy. These findings highlight the underuse of gastroprotection in a substantial proportion of patients, despite their high bleeding risk and concomitant antithrombotic treatment. This represents a missed opportunity for prevention, as current guidelines recommend routine gastroprotection in high-risk patients treated with antithrombotics [10,11]. Improving adherence to these recommendations could substantially reduce bleeding-related hospitalizations.

Decisions regarding the resumption of antithrombotic therapy after bleeding remain particularly challenging. In this snapshot study, one-third of patients with AF were discharged without any antithrombotics. Our data do not imply inappropriate care. Instead, they highlight uncertainty and risk-averse decision-making. Although consensus documents provide general guidance, evidence-based recommendations on the optimal timing and choice of therapy are limited. The 2020 American College of Cardiology Expert Consensus Pathway emphasizes an individualized approach based on multidisciplinary evaluation of thrombotic and bleeding risks, also taking into consideration the site of bleeding and the risk of rebleeding before resuming therapy [12]. Similarly, the 2024 European Society of Cardiology guidelines for the management of AF highlight the importance of intensifying efforts to modify existing risk factors and carefully reviewing the choice of OAC after a bleeding episode in patients treated with OACs [9]. In this context, potentially modifiable bleeding risk factors, such as uncontrolled hypertension, diabetes, kidney disease, alcohol consumption, and potential drug–drug interactions, should be systematically identified and addressed.

NOACs are associated with a lower bleeding risk than warfarin and should be preferred in patients with a labile international normalized ratio or when combination therapy is required [9,13]. Reduced-dose NOAC regimens, when used according to label-recommended criteria, maintain efficacy while lowering bleeding risk; however, inappropriate dose reduction should be avoided. Although switching between NOACs is common, particularly favoring apixaban after GI bleeding, robust supporting evidence is limited [14]. Notably, a significant proportion of patients in this snapshot study were discharged on LMWH, despite the absence of evidence supporting its long-term use for stroke prevention in AF. This observation may reflect clinician hesitation to restart oral anticoagulation after a major bleeding event and underscores a potential gap between guideline recommendations and real-world practice. Regarding antiplatelet therapy, prolonged use beyond guideline-recommended durations, especially in patients already receiving oral anticoagulation, should be avoided [11].

AF was the indication for antithrombotic treatment in nearly half (46.2%) of patients experiencing a hemorrhagic event, whereas relatively few had a strict indication for vitamin K antagonists. While OACs remain the standard of care for stroke protection in AF**,** alternative options should be considered for patients at high bleeding risk. In this context, transcatheter LAA closure offers effective thromboembolic protection with a lower bleeding risk and may be appropriate for a substantial proportion of patients hospitalized with bleeding [15]. Increased awareness among internal medicine physicians is essential to ensure timely referral of eligible patients.

Several patient-related factors are known to increase the risk of bleeding in individuals treated with antithrombotic medication, including advanced age, uncontrolled hypertension, prior bleeding or stroke, and comorbidities such as chronic liver or kidney disease. Frailty has also been recognized as an independent predictor of bleeding risk, with higher levels of frailty associated with increased risk [16]. Patients in this study were elderly (mean age, 79.5 years) with moderate frailty (mean Rockwood Frailty Scale score, 4.8). Bleeding occurred even in patients who were not severely frail, likely reflecting greater exposure to antithrombotic therapy in this group. Importantly, frailty was the only factor independently associated with mortality, highlighting its value in prognostic assessment and the need for intensified management in frail patients experiencing major bleeding [17,18].

The strengths of this study include its multicenter design and real-world snapshot approach, providing an estimate of the burden of bleeding-related hospitalizations. However, limitations include the cross-sectional design, lack of long-term follow-up, lack of a comparator cohort without bleeding, and the use of hospitalization-based rather than standardized bleeding definitions. More specifically, by including all patients hospitalized for clinically significant bleeding rather than restricting eligibility to ISTH-defined major bleeding events, patients with bleeding episodes that would not meet major bleeding criteria may have been enrolled in our study. While this pragmatic approach was designed to capture real-world clinical practice, it may reduce the comparability of our findings with those of studies that exclusively evaluate ISTH-defined major bleeding. Another limitation is that the short enrollment period may not fully capture seasonal or annual trends. Consequently, we cannot determine whether such trends exist or to what extent they may affect the generalizability of our results. Moreover, the cross-sectional snapshot design does not allow assessment of outcomes associated with different antithrombotic management strategies. In particular, no follow-up was performed; thus, evaluation of subsequent thromboembolic or bleeding events after hospital discharge was not possible. As a result, the clinical consequences of the observed treatment approaches cannot be determined.

## 5 Conclusions

Major bleeding events in patients receiving antithrombotic therapy represent a substantial clinical challenge, imposing a significant inpatient burden across internal medicine and gastroenterology departments and are associated with high morbidity and mortality. Postbleeding antithrombotic management remains complex and heterogeneous and should be guided by an individualized approach that carefully balances thrombotic and bleeding risks, supported by shared clinician-patient decision-making. In patients with AF, who account for a large proportion of bleeding events, alternative strategies that offer effective thromboembolic protection, particularly LAA occlusion, should be considered when long-term anticoagulation is not feasible.

## Appendix

**Contributing Centers and Investigators.**

**ALEXANDRA HOSPITAL Department of Clinical Therapeutics**: M. Dimopoulos, M. Gavriatopoulou, A. Mitrakou; **First Internal Medicine Department:** D. Sinapidis, E. Kostis, E. Kerezoudi; **GI Unit**: S. Michopoulos; **ATTIKO HOSPITAL Fourth v of Internal Medicine:** N. Kontomitros; **Second Propaedeutic Department of Medicine**: S. Pililis; **ELPIS HOSPITAL Internal Medicine Department**: A. Fragkou, S. Lagou, L. Lemonidis, K. Vrysis; **GI Unit:** P. Kalisperati; **EVANGELISMOS HOSPITAL First Internal Medicine Department**: T. Kontopoulou, I. Iliopoulos, G. Pournaras; **Third Internal Medicine Department:** V. Sevastianos, P. Stoikos, E. Geladari; **Fourth Internal Medicine Department:** G. Markomichelakis, P. Efthimiou, D. Katsavarou; **Fifth Internal Medicine Department:** V. Papastamopoulos, A. Siampanos, N. Petridis, M. Chroni; **GI Unit:** N. Viazis; **GENNIMATAS HOPSITAL First Internal Medicine Department**: G. Adamis; **Second Internal Medicine Department**: A. Touliatou, C. Valilas, A. Kougioumtzoglou, G. Grigoriou, E. Tzintziloni; **Third Internal Medicine Department:** S. Pagoni; **GI Unit:** I. Karoumpalis; **GENERAL ONCOLOGICAL HOSPITAL OF KIFISIA**: **Internal Medicine Department:** G. Boulmetis, A. Argyriadi, G. Kourmpelis; **Second Oncology Unit:** G. Aravantinos; **HIPPOKRATION HOSPITAL Second Internal Medicine Department**: I. Mani; **“KAT” GENERAL HOSPITAL Internal Medicine Department**: I. Kyriazis, A. Apostolos; **KONSTANTOPOULEIO HOSPITAL First Internal Medicine Department**: C. Lampropoulos, E. Koukou, V. Argiana, S. Zacharioudaki; **Second Internal Medicine Department:** A. Tzavaras, E. Kaldara, K. Katsiofoti; **GI Unit:** G. Tziatzios; **LAIKO HOSPITAL First Department of Propaedeutic and Internal Medicine**: P. Sfikakis, E. Apostolopoulou; **First internal Medicine Department:** E. Gkogka, M. Samarkos; **Pathophysiology Department:** M. Voulgarelis, E. Apostolidi, D. Germanou; **NIKAIA GENERAL HOSPITAL Third Internal Medicine Department:** E. Michelakis, A. Vakka, S. Gaitanou, S. Fragkiski, S. Menegakis, E. Michaliou, A. Liapi; **PAMAKARISTOS HOSPITAL Internal Medicine Department**: E. Agelonidou, K. Souvatzi, A. Krilis, E. Diamantaki, A. M. Xirikou; **RED CROSS HOSPITAL First Internal Medicine Department**: V. Tzavara, A. Lioni, V. Lampropoulou, D. Papanikolaou; **Third Internal Medicine Department:** M. Chini, D. Kostourou, E. Manou, E. Geroniti; **GI Unit**: G. Trimponias, P. Zormpas, M. Spinou; **SISMANOGLIO HOSPITAL First Internal Medicine Department**: E. Tzivaki; **Second Internal Medicine Department:** M. Lada, V. Kolonia, M. Mprinia; **GI Unit:** E. Lalla; **SOTIRIA HOSPITAL Third Internal Medicine Department:** K. Syrigos, A. Kollias; **First Internal Medicine Department**: A. Argyraki, T. Katsikas, C. Zirou**; GI Unit** G. Mpamias, C. Gkizis, I. Koutsounas; **TZANIO HOSPITAL First Internal Medicine Department**: S. Antonopoulos, G. Athanasakis, M. Vroucho; **THRIASIO HOSPITAL First Internal Medicine Department**: S. Sympardi, E. Kaltsounis, T. Tsampras; **Second Internal Medicine Department:** K. Iliopoulou; **401 MILITARY HOSPITAL Second Internal Medicine Department**: N. Papadopoulos, G. Iliopoulos, A. Katsiki, M. Mpoulougari.
